# Enhancement in Post-Consumer Mechanical Recycling of Plastics: Role of Design for Recycling, Specifications, and Efficient Sorting of Packaging Material

**DOI:** 10.3390/polym17091177

**Published:** 2025-04-25

**Authors:** Thomas Rumetshofer, Jörg Fischer

**Affiliations:** Institute of Polymeric Materials and Testing, Johannes Kepler University Linz, Altenberger Straße 69, 4040 Linz, Austria

**Keywords:** digitalization, circular economy, sustainability, plastics recycling

## Abstract

Plastic packaging materials can play a significant role in turning the plastic industry towards a circular economy, owing to their large volumes and short product lifetimes. This study emphasizes the role and interaction of design for recycling (DfR), appropriate specifications, and efficient sorting. DfR is enhancing the recyclability of plastic packaging by selecting appropriate materials and designs, improving the quality of recyclates without compromising safety or the original requirement. A significant barrier to achieving a circular economy is the lack of comprehensive standards for recycled plastics. While some specifications exist, a more integrated and globally accepted standardization regime, similar to that in the aerospace industry, is necessary to ensure quality and consistency in recycled materials. The potential of advanced sorting technologies to improve sorting efficiency and feedstock quality is highlighted, significantly enhancing recovery yields and the quality of recyclates. Information-based tracking technologies, such as digital watermarks, offer substantial benefits in identifying and sorting materials with high granularity, improving sorting mechanisms, enhancing resource recovery, and providing valuable data for stakeholders across the plastic value chain. The implementation of information-based technologies can reduce production costs and environmental impacts, with exemplary calculations indicating a potential 30% reduction in the production cost of PP recyclate.

## 1. Introduction

**Figure 1:**
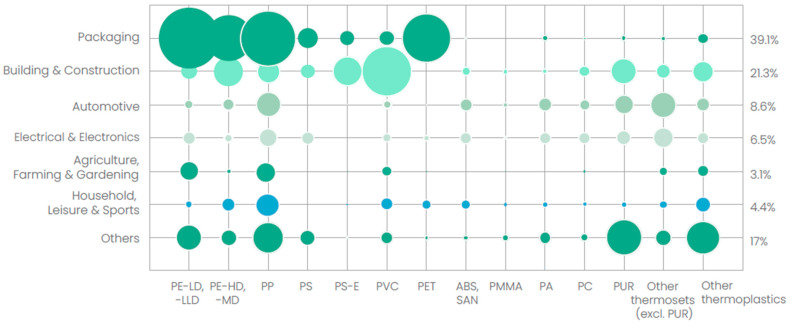
European plastic demand by application and type [1].

In 2021, global plastics production, including post-consumer recycled and bio-based plastics, was 390.7 million tons, with a share of 57.2 million tons for European production [1]. The largest portion of this volume was attributed to packaging, with a share of 39.1% by weight, as illustrated in Figure 1. To achieve predetermined requirements, the packaging industry mainly uses low-density polyethylene (PE-LD), linear low-density polyethylene (PE-LLD), high-density polyethylene (PE-HD), polypropylene (PP), and polyethylene terephthalate (PET) as base materials, often combined with functionalized layers to provide a barrier against microorganisms, oxygen, moisture, and light [2].

A total of 5.5 million tons of post-consumer recycled plastics was reintroduced into Europe’s plastic market in 2021, representing approximately 10% of the plastics converted in the European Union [1]. Most of this volume does not end up in packaging applications because of the materials’ performance and legal restrictions on food safety [3]. On the other hand, 17.9 million tons of post-consumer packaging waste (mixed and separate collection) were collected in 2020 across Europe, representing a sizable input stream for plastic recycling facilities [1].

**Figure 2:**
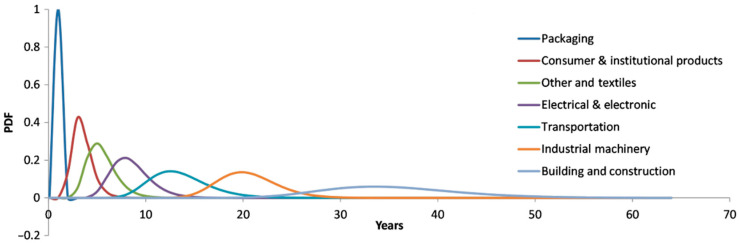
Product lifetime distributions for the eight industrial use sectors plotted as log-normal probability distribution functions (PDFs) [4].

In addition to recyclability, the lifetime of a plastic product is another important parameter that needs to be considered. Figure 2 shows the product lifetime distribution for eight different industrial sectors, identifying packaging as the sector with the shortest lifetime of less than one year [4].

The pressure on the packaging industry to provide suitable solutions for a circular economy comes from multiple perspectives. Major brand owners will voluntarily pledge the use of recycled materials in packaging in the coming years. Pledges of more than 1000 companies and organizations in the packaging area are combined in Ellen McArthur’s Global Commitment with the aim of increasing recyclability to 100% and the use of recycled material to 25% by 2025 [5]. Kahlert et al. [6] stated that pledges can help stimulate circularity and investments but are not suitable for solving all problems. On top of this, politicians of the European Union started to realize the problems around plastic materials and started to set actions to mitigate the negative impact of plastics. The European Action Plan [7] for a circular economy dealing with marine litter, hazardous chemical additives, and the recycling of plastic packaging was introduced in 2015. As a framework for driving innovation, investments, and circular solutions, a European strategy for plastics [8] was brought up in 2018. One policy area of this framework is plastic materials, with the aim of tackling plastic pollution and marine litter. Based on this, specific directives and policies were adopted to achieve these goals.

- The Packaging and Packaging Waste Directive (94/62/EG), with its amendment in 2018, aims to harmonize the management of packaging and packaging waste within the European Union. It declares a recycling rate of at least 50% by the end of 2025 and 55% by the end of 2030 as targets and defines a hierarchy of waste (reduce, reuse, recycle, and dispose) [9];
- European Directive (2008/98) and its amendment in 2018 on waste management, extended producer responsibility (EPR), and waste prevention [10];
- European Directive (EU/2015/720) to reduce the consumption of lightweight plastic carrier bags to prevent the littering of plastic carrier bags and other items into the environment [11];
- European Directive (EU/2019/904) to reduce the impact of certain plastic products on the environment. This directive restricts the consumption of single-use plastic (SUP) items in Europe [12];
- The regulation on the shipment of waste (EC/1013/2006), amended at the Basel Convention in 2020, has the primary objective of protecting the environment in international trade [13,14];
- Commission Regulation (EU/2022/1616) defines the rules and requirements for recycled plastic materials and articles intended to come into contact with food [15].

Several of the abovementioned directives and regulations follow the target to establish a circular economy and increase recycling rates in Europe. Recycling rates in Europe are influenced on the one hand by the calculation methods used and by different types of technological and social losses, mainly affecting the output-related recycling calculation method [16]:

- Collection rate: accumulated losses due to incorrect sorting, streams towards incineration or landfill, and littering towards the environment;
- Sorting rate: material loss during sorting process in waste handling and recycling facilities;
- Recycling process rate: losses, off-specification, and waste production during recycling processes within recycling plants.

Many mechanical recycling processes, especially in non-sensitive applications, are already optimized towards product losses, because efficiency improvements help to optimize production costs. Many initiatives and public awareness programs are ongoing to create awareness about resource recovery and increase plastic collection rates [17]. Material loss during the sorting process, which can happen through customers and waste collectors within pre-sorting, sorting, or recycling facilities, is widespread and still a problem.

## 2. Methodology

The primary aim of this study is to demonstrate that a robust integration of Design for Recycling (DfR), appropriate material specifications, and efficient sorting processes is essential for transitioning the plastics industry towards a circular economy. This study analyzes DfR approaches from the literature to identify design elements that facilitate or hinder recycling and product designs that enhance recyclability without compromising functionality. For specifications, we will investigate frameworks to ensure compliance with regulatory standards and market needs, drawing parallels from industries such as aerospace. For the sorting process, automated techniques and non-destructive spectroscopic methods are investigated from the literature. Additionally, advanced sorting technologies, including the implementation of information-based tracking technologies such as digital watermarks, are evaluated to improve material identification and sorting granularity.

## 3. Design for Recycling (DfR)

Plastic packaging materials have a huge impact on the plastics industry owing to their high volume and short life cycles. Flexible packaging applications are one example that face challenges with current designs in terms of circularity. To achieve the required properties, PET is often used as a barrier layer for PE-based packaging, which is immiscible with base materials and created together with inks, labels, additives, and fillers in recycling processes and which lowers the product quality of recyclates [18]. Schlossnikl et al. [19] reported that even a small fraction of unwanted components can cause a noticeable deterioration in the final product quality. Design for Recycling (DfR) for packaging is an approach to find solutions that reduce the environmental impact of the product’s life cycle with a focus on the end-of-life system, low impact of materials, and proper material selection without compromising safety or the original packaging requirements [20]. Numerous academic institutions and associations have provided designs for recycling guidelines.

RecyClass [21] developed design for recycling guidelines and an online simulation tool to obtain insights into the compatibility of different packaging elements, such as caps, labels, or adhesives, with given recycling streams. To make flexible packaging circular, the circular economy for flexible packaging Europe initiative (CEFLEX) [22] created a circular economy guideline with principles and information to help deliver significant environmental improvements without compromising the functionality to protect, package, transport, sell, or use the product. The Ellen McArthur Foundation [23] initiated in 2017 the circular design guide, which offers a number of activities to understand circular economy design, define suitable circular business models, take circular concepts forward, and release them through partnerships. The Waste and Resources Action Programme (WRAP) [24] published a guideline focusing on rigid packaging for household goods, which provides practical guidance for the preferred materials and formats. Tacker et al. [25] developed in 2020, together with FH Campus Wien [26], circular packaging design guidelines for different packaging systems, with material selection, decoration aspects, and closure systems as key execution features. Eco Design for plastic packaging [27] provides guidelines and strategies for optimized resource needs, sustainable sourcing, design for environmentally sound use, and design for recycling. The Netherlands Institute for Sustainable Packaging (KIDV) [28] published a roadmap to show strategies and gaps to bring multi-layer flexible packaging material into a circular economy. The University of Antwerp [29] followed a different approach and developed a guideline for design from recycling, which investigated the extent to which new products can be manufactured from an existing recycling flow and which product design specifications are necessary.

At the moment, there is only a limited number of packaging designed according to these guidelines, but packaging design according to DfR will become increasingly relevant in future [30]. Seier et al. [31], for example, investigated in a case study a recyclable, mono-material modified atmosphere packaging (MAP) with PP as the base material and found that the packaging was recyclable and maintained its mechanical properties and barrier functionality towards oxygen and moisture. Carullo et al. [32] compared a coated PE-based mono-material packaging solution with three market solutions and found outstanding performance in terms of oxygen and water barrier properties. Maga et al. [33] studied the environmental impact of various packaging solutions for meat trays and reported that multi-layer products exhibited higher environmental impacts.

Overall, it can be concluded that there are many initiatives on DfR, but the current waste streams are still not optimal for recycling plastic packaging waste. The aim of the DfR is to provide clean mono-material streams in the future to enable high-quality recycling for appropriate end applications.

## 4. Specification

Standardization and specifications have been established for decades and represent tools to ensure quality and consistency in the plastics industry. Numerous standards for materials and applications are available in virgin plastic businesses. As the plastic recycling industry is an emerging business that must deal with variation from multiple sides, no highly differentiated standardization regime has been established until now. Nevertheless, some specific specifications regarding plastic recyclates and their characterization are already available. German Institute for Standardization (DIN) specification 91,446 from 2021 [34] classifies recycled plastics by Data Quality Levels (DQLs) for use and (digital) trading. This standard defines DQLs based on the available data depth, provides guidelines for labeling, and provides guidance for the characterization of plastic waste as a feedstock material. DIN specification 91,009 from 2015 [35] deals with the determination of marker compounds in food-grade recycled PET materials and provides detailed information and guidelines on quality control methods. DIN specification 91,011 from 2013 [36] provides details on sample preparation for recycled plastics for mechanical and chemical analyses.

Notwithstanding the abovementioned specifications, standards for market applications made from recycled materials are missing, and the framework needs to be more comprehensive, focusing on high quality plastic materials. If this is fulfilled, standards can play a significant role in establishing a circular economy, as the PET bottle industry has demonstrated its risk assessments and sophisticated super-clean processes over the last two decades [37]. One area where the plastics industry can learn is aerospace standardization, which is a highly integrated enabler to achieve technical excellence, is globally accepted, and serves a leadership role within the standardization organizations [38]. The aerospace industry is always heading for globally harmonized, technically optimized, and completely integrated standards. However, this system is very complex and time-consuming, generates additional effort, and faces implementation restrictions. The plastics industry can learn from best practices in the aerospace area and can use standards and certification schemes in a broader sense for handovers between the different actors in the plastic value chain.

## 5. Sorting Improvements

An industrial recycling facility for the mechanical recycling of plastic waste currently contains the main technology steps of sorting, grinding, washing, and extrusion, whereas for the sorting of mixed plastic fractions, manual sorting by trained operators or automated sorting by sensor-based sorting (SBS) on a sorting belt are used [39]. Due to its diverse composition and remaining residues, the processing of packaging waste requires more attention and additional steps in sorting. After the first shredding step, fines are removed by a sieving step (e.g., arotating drum sieve), followed by density-based float–sink separation and mass-based air classifier separation at the flake level [39]. Numerous strategic and technological improvements have been proposed in the literature to increase sorting efficiency in plastic recycling plants.

Neo et al. [40] presented a comprehensive overview of recent developments and improvements in non-destructive spectroscopic methods for sorting and highlighted their significant potential. Infrared spectroscopy (IR), Raman spectroscopy, laser-induced breakdown spectroscopy (LIBS), and hybrid systems were analyzed. Hybrid spectroscopic methods, combined with the development of an open-source database and the use of novel machine learning tools, offer the highest potential to overcome the limitations of global plastic waste sorting.

Lase et al. [2] conducted a material flow analysis on a quality recycling process (QRP) proposed by CEFLEX, which focuses on polyolefin flexible packaging and involves five additional NIR-based sorting steps with specific target products. Although a QRP offers a similar overall process yield compared to standard mechanical recycling processes, the quality of the produced recyclates and recovery yields of the targeted products are significantly improved.

In addition to improvements in the currently used technologies, new and disruptive technologies are being pushed on the market. One example of these new technologies is sorting using image or object recognition in combination with artificial intelligence (AI) and picking robots. Sarc et al. [41] provided a comprehensive overview of robotics-based sorting systems, with several examples and their limitations. Wilts et al. [42] reported opportunities for digitalization in waste management and sorting tests using the ZenRobotics test system. Konstantinidis et al. [43] designed a cyber–physical sorting system (CPSS) that consists of an optical camera to track the position, a combined hyperspectral and short-wave infrared (SWIR) sensor to identify the item, a deep learning model, and a robotic arm to separate the items from the conveyor belt. Heikkilä et al. [44] conducted an interview-based study on circular value creation and found that smart robots can add significant value for sorting, owing to an improved output quality and higher efficiency.

A practical example of the importance of sorting is PET bottles, where a value stream has been established within the last decades, and in many countries, separate sorting streams exist. In 2019, the collection rate of PET bottles in Austria reached 76% of all bottles brought to the market, with a high sorting depth [45]. Within the same timeframe, the collection rate for all plastic packaging materials reached only 25%. Although PET bottles represent a waste stream with low complexity, additional effort, with an increase in the collection rate to 90% and a sorting depth of at least 80%, is needed until 2029 to fulfill EU targets.

## 6. Information-Based Technologies

With improved sorting technologies, higher sorting efficiencies can be achieved; however, one can only determine what is available in the feedstock. Another approach to increase sorting efficiency and feedstock quality, in order for the plastics industry to become a circular economy, is the use of information-based tracking technologies. There are a variety of different pathways for information-based tracking systems in the literature and in the market [46]. In this study, we focus on one example in the field of physical tracking.

**Figure 3:**
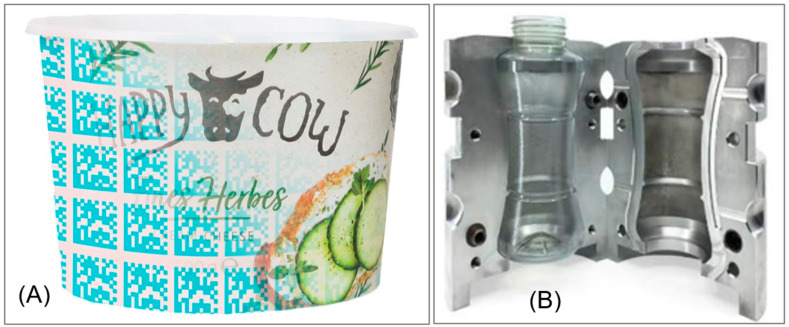
Imperceptible digital watermarks in (**A**) printed tiles and (**B**) mould variations; image courtesy of P&G/Digimarc/Logoplaste/HolyGrail 2.0 [48].

HolyGrail 2.0 [47] is a cross-value chain initiative driven by the European Brands Association and powered by the Alliance to End Plastic Waste, gathering over 120 companies and organizations from the packaging value chain with the objective of proving the viability of digital watermarking technologies for accurate sorting and the business case at a large scale. Digital watermarks are imperceptible optical codes with the size of a postage stamp that can carry a wide range of attributes or can be linked to a digital product passport (DPP). As this coding technology is invisible, repeated tiles printed on packaging or repeated micro-topological variations embedded into molds create a signal that carries the information, as shown in Figure 3 [48]. The project focuses on intelligent sorting with an add-on module for existing sorters, data mining along the value chain, and increasing consumer engagement. In a semi-industrial trial with 125,000 packaging samples, the consortium achieved detection and purity rates of 99% and 95%, respectively, across all tested categories.

The focus and strength of digital watermarks are the identification of materials and substrates with ease, reliability, and high granularity during the recycling process and the improvement of sorting mechanisms. In addition to improvements in recycling, digital watermarks also show benefits in manufacturing, logistics, and distribution centers to verify the material flow, can assist in verification during the sales or check-out process, and can improve the customer experience during sales and at home in manifold ways [48].

However, the advantages shown for information-based tracking technologies require additional effort and have certain drawbacks. First of all, additional equipment and detection capabilities are needed in every sorting facilities, and digital watermarks cannot detect the misuse of packaging materials, mingling, or residues. Digital watermarks focus on the final product and cannot support intermediate production process steps [49]. In the market, it can only work with broad acceptance, and open questions around data ownership and data security are not clarified appropriately [46].

## 7. Benefits of Use of Information-Based Technology and DfR

Jensen et al. [50] investigated in an interview case study the different needs of seven stakeholder groups along the mechatronics value chain and identified differentiating needs for data, the exchange of data, and supporting infrastructure as major bottlenecks. Nordic Innovation [51] explored the enhancement of existing business models towards more sustainability and the establishment of completely new business models as the major advantages for data sharing in a circular economy.

**Figure 4:**
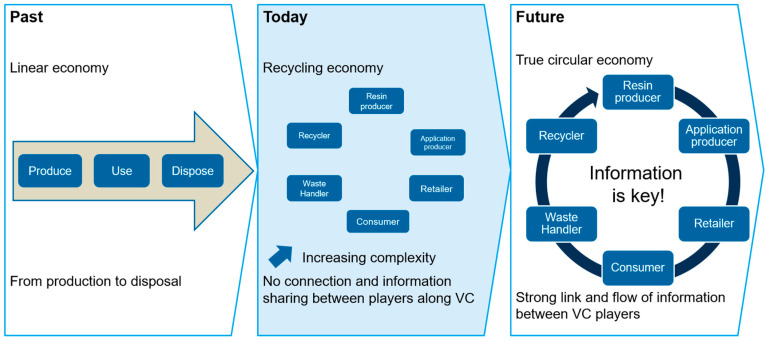
Past, current, and future model for the plastics industry, reprinted from [46].

Rumetshofer et al. [46] illustrated that the flow of information between different players is a key factor for a future circular economy in the plastics industry. Figure 4 presents the past, current, and future models in the plastics industry and underlines the connection between the different players in the plastic value chain. In a future information-based circular economy, everyone should have an advantage from the use of disruptive information technologies and DfR packaging designs.

Resin and application producers can profit from the availability of high-quality recyclates [2,52], which helps fulfill mandatory legislative requirements [9], and the credible, secure traceability of material streams [53]. Retailers can benefit from these advantages by enhancing customer engagement [54] and the ability to transfer information more efficiently to consumers [51]. Information technologies and DfR can help retailers fulfill their voluntary pledges announced by 2025 [5]. Consumers can gain an advantage by receiving more information on the product or packaging, increased product safety, reliable origin declaration [55] or an extension of the product use phase [51].

Waste handlers can profit from the implementation of information technologies and DfR through a decreased effort for sorting with a higher quality [44], additional information on packaging materials, more efficient resource recovery [51], and a higher sales revenue. Recyclers can profit from an increased input quality, which leads to an increased product quality, fewer residues in the recycling process that need to be burned [56], and lower production costs.

The mechanical recycling of different types of polymers shows benefits by reducing the environmental burden, and the use of information-based technologies decreases the production costs of recyclates. To quantify the environmental impact of different packaging alternatives, life cycle assessment (LCA) is an appropriate and well-established tool. Bher et al. [57] conducted a meta-analysis of 131 LCA studies on packaging systems and assessed their adherence to the standard requirements. It was found that LCAs are of utmost importance to foster confidence in conducting fair comparisons and progress toward sustainable development goals. Perugini et al. [58] compared five alternative plastic waste management options and concluded that the mechanical recycling scenario showed a 70–80% decrease in emissions of greenhouse gases (GHGs) with respect to the non-recycling scenarios.

In the following, we show the decrease in production costs for PP at the recycler along with the implementation of disruptive information-based technologies. As a basis, the production of 1 t of PP recyclate was assumed (base case scenario in Table 1). To reflect the benefit for the waste handler, a 10% increase in the feedstock with the use of novel sorting technology was considered (advanced case scenario in Table 2).

In Europe, 10.2 Mt of post-consumer plastic material was collected and recycled in 2020 [1]. At the same time, only 5.5 Mt of post-consumer recyclate (PCR) products have been introduced into the market. This leads to an average yield of 54% across all polymer types, when imports and exports are neglected. As an average price for scraped, pre-sorted PP in bale form, 6.5 US cents per pound (cost basis June 2024) can be taken into account [59]. This corresponds to a 144 USD per metric ton of feedstock cost in the base case. The reprocessing of pre-sorted plastic material contributes 306 USD/t to the overall cost, and incineration adds another 170 USD per ton of reject material [56].

Table 1 presents the base case scenario for the production of 1 t PP recyclate with a 306 USD/t production cost per ton of feedstock, an average yield of 54%, and the assumption that the remaining feedstock is incinerated. This adds up to production costs of approximately 979 USD/t for the PP recyclate produced.

Over the long term, novel information-based technologies and DfR should increase the feedstock quality and bring the yield closer to the feedstock specification of 94% PP in the case of a DSD 324-0 bale [60]. For the advanced case, we assumed to close the gap between the current situation and the specification halfway, resulting in an improved average yield of 74%. However, the increased quality of sorted PP bales can lead to higher sales prices for the sorting of plastic waste. Here, we assumed an increase of 10%, which leads to an increased feedstock cost of 158 USD/t.

Table 2 shows the advanced case scenario for the production of 1 t PP recyclate with an advanced yield of 74%. This leads to a production cost of approximately 687 USD/t of PP recyclate. Overall, this exemplary calculation indicates that the total recyclate production cost could potentially be reduced by 30% for the advanced case and 292 USD/t for the PP recyclate produced

## 8. Discussion

**Figure 5:**
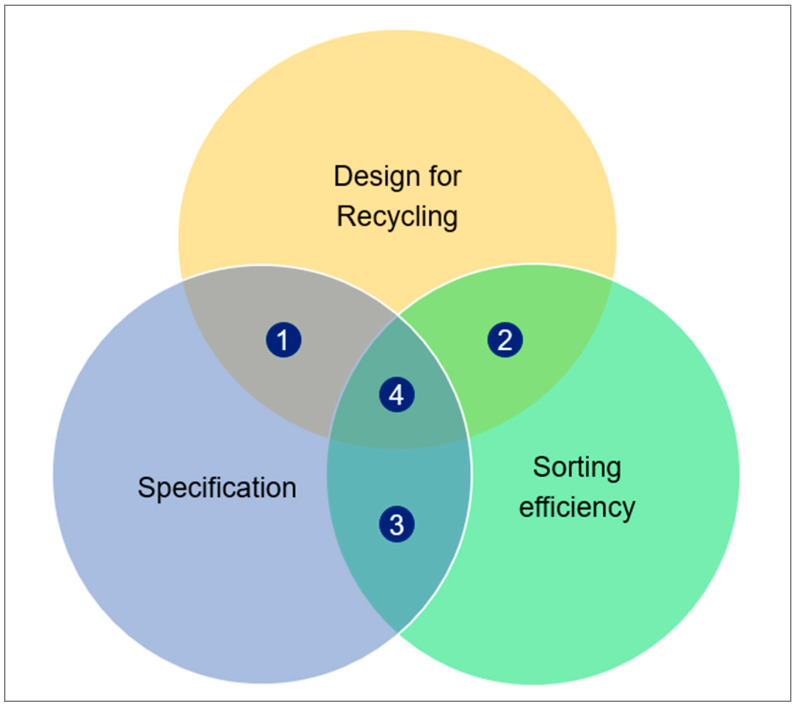
Overlapping areas of DfR, specification, and sorting efficiency.

It has been shown that specifications, design for recycling, and efficient sorting can contribute in their respective areas to shifting the plastic industry towards a circular economy. Figure 5 highlights possible overlaps between these three areas. If only specification and DfR (area 1) are connected, the circular economy cannot work, because there is not enough material for recycling available, specifications are not met because of insufficient sorting quality, and the overall recycling process is economically unattractive, as shown in the example above. On the other hand, it is not sufficient to have a proper design for recycling and efficient sorting (area 2), because the material cannot be used in more demanding applications if it does not fulfill the requirements and production needs, which leads to the downcycling of high-quality input material. It is also futile to have efficient sorting and proper specifications at hand (area 3), because without a suitable DfR, there is not enough high-quality material available that can be sorted adequately and reach the required quality.

From our point of view, a robust and seamless combination of DfR, specification, and efficient sorting (area 4) can reach the goal and turn the plastics industry into a circular economy. This combination reflects a sweet spot, and each area needs to contribute. An adequate DfR ensures that all plastic materials entering the market can be recycled after their intended use and generates a high-quality material stream for recycling. Specifications for plastic materials and applications that consider the origin and history of recyclates need to provide quality criteria that ensure that recycled material can stay in the loop in similar applications. With efficient sorting and disruptive information-based technologies, waste handlers can easily identify useful materials from various waste streams and provide high-quality material streams.

The concept of a combination of DfR, specification, and proper sorting sounds simple and easy to implement, but there are a few hurdles and weaknesses that need to be considered. Efficient sorting, particularly digital information-based tracking technologies, can generate arbitrary fractions regarding material type, formulation, color, or size.

## 9. Conclusions

This study emphasizes the crucial role of design for recycling in enhancing the recyclability of plastic packaging by selecting appropriate materials and designs, which can significantly improve the quality of recyclates without compromising safety or the original requirements. Future packaging designs must increasingly follow DfR guidelines to ensure clean mono-material streams for high-quality recycling. A significant barrier to achieving a circular economy is the lack of comprehensive standards for recycled plastics. While some specifications exist, a more integrated and globally accepted standardization regime, similar to that in the aerospace industry, is necessary to ensure quality and consistency in recycled materials and facilitate their use in demanding applications. This study highlights the potential of advanced sorting technologies, including non-destructive spectroscopic methods and AI-driven systems, to improve sorting efficiency and feedstock quality, significantly enhancing the recovery yields and quality of recyclates, contributing to a more efficient recycling process.

Information-based tracking technologies, such as digital watermarks, offer substantial benefits in identifying and sorting materials with high granularity, improving sorting mechanisms, enhancing resource recovery, and providing valuable data for stakeholders across the plastic value chain, though their implementation requires additional equipment and broad market acceptance. The implementation of advanced sorting and information-based technologies can reduce production costs and environmental impacts, with exemplary calculations indicating a potential 30% reduction in the production cost of PP recyclate. Additionally, life cycle assessments show that mechanical recycling scenarios significantly decrease greenhouse gas emissions compared to non-recycling scenarios.

Although there are open questions and still a way to go, we believe that a seamless combination of DfR, proper standardization, and efficient sorting is essential to transition the plastics industry towards a circular economy, with each component playing a crucial role: DfR ensures recyclability, standardization provides quality criteria, and efficient sorting enables the identification and recovery of high-quality materials. Overcoming the hurdles in implementing these components will be key to achieving a sustainable and economically viable circular economy.

## Figures and Tables

**Table 1:** Base case scenario for production of 1 t PP recyclate.

Base Case Scenario		
Feedstock cost	144	USD/t
Production cost	306	USD/t
Average yield	54	%
Incineration cost	170	USD/t
Cost for 1 t of recyclate	979	USD/t

**Table 2:** Advanced case scenario for production of 1 t PP recyclate.

Advanced Case Scenario		
Feedstock cost	158	USD/t
Production cost	306	USD/t
Average yield	74	%
Incineration cost	170	USD/t
Cost for 1 t of recyclate	687	USD/t

## Data Availability

The data presented in this study are available upon request from the corresponding author.
